# Block Copolymer-Based Magnetic Mixed Matrix Membranes—Effect of Magnetic Field on Protein Permeation and Membrane Fouling

**DOI:** 10.3390/membranes11020105

**Published:** 2021-02-02

**Authors:** Lakshmeesha Upadhyaya, Mona Semsarilar, Damien Quemener, Rodrigo Fernández-Pacheco, Gema Martinez, Isabel M. Coelhoso, Suzana P. Nunes, João G. Crespo, Reyes Mallada, Carla A. M. Portugal

**Affiliations:** 1 Advanced Membranes and Porous Materials Center (AMPM), Biological and Environmental Science and Engineering Division (BESE), King Abdullah University of Science and Technology (KAUST), 23955-6900 Thuwal, Saudi Arabia; lakshmeesha.upadhyaya@kaust.edu.sa (L.U.); suzana.nunes@kaust.edu.sa (S.P.N.); 2 Institut Européen des Membranes, IEM UMR 5635, Univ Montpellier, ENSCM, CNRS, 34070 Montpellier, France; mona.semsarilar@umontpellier.fr (M.S.); damien.quemener@umontpellier.fr (D.Q.); 3 Laboratorio de Microscopías Avanzadas (LMA), Instituto de Nanociencia y Materiales de Aragón (INMA), CSIC-Universidad de Zaragoza, 50018 Zaragoza, Spain; pacheco@unizar.es; 4 Networking Research Centre on Bioengineering, Biomaterials and Nanomedicine, CIBER-BBN, 28029 Madrid, Spain; gemamar@unizar.es; 5 Instituto de Nanociencia y Materiales de Aragoń (INMA), CSIC-Universidad de Zaragoza, 50009 Zaragoza, Spain; 6 LAQV-REQUIMTE, Departamento de Química, Campus de Caparica, Faculdade de Ciências e Tecnologia, Universidade Nova de Lisboa, 2829-516 Caparica, Portugal; imrc@fct.unl.pt (I.M.C.); jgc@fct.unl.pt (J.G.C.)

**Keywords:** block copolymers, magneto-responsive membranes, superparamagnetic nanoparticles, protein filtration

## Abstract

In this study, we report the impact of the magnetic field on protein permeability through magnetic-responsive, block copolymer, nanocomposite membranes with hydrophilic and hydrophobic characters. The hydrophilic nanocomposite membranes were composed of spherical polymeric nanoparticles (NPs) synthesized through polymerization-induced self-assembly (PISA) with iron oxide NPs coated with quaternized poly(2-dimethylamino)ethyl methacrylate. The hydrophobic nanocomposite membranes were prepared via nonsolvent-induced phase separation (NIPS) containing poly (methacrylic acid) and meso-2,3-dimercaptosuccinic acid-coated superparamagnetic nanoparticles (SPNPs). The permeation experiments were carried out using bovine serum albumin (BSA) as the model solute, in the absence of the magnetic field and under permanent and cyclic magnetic field conditions OFF/ON (strategy 1) and ON/OFF (strategy 2). It was observed that the magnetic field led to a lower reduction in the permeate fluxes of magnetic-responsive membranes during BSA permeation, regardless of the magnetic field strategy used, than that obtained in the absence of the magnetic field. Nevertheless, a comparative analysis of the effect caused by the two cyclic magnetic field strategies showed that strategy 2 allowed for a lower reduction of the original permeate fluxes during BSA permeation and higher protein sieving coefficients. Overall, these novel magneto-responsive block copolymer nanocomposite membranes proved to be competent in mitigating biofouling phenomena in bioseparation processes.

## 1. Introduction

Membrane technology is an efficient method for the separation of macromolecules such as proteins, fibers, and many other compounds [1,2]. A significant drawback of membrane technology results from the presence of fouling pheomena caused by the accumulation of solutes on the membrane surface or within the membrane porous structure [3,4]. Membrane fouling not only affects the performance of the membranes irreversibly but also increases the overall operational cost [5,6] due to the need of regular membrane cleaning procedures. To date, extensive research has been carried out involving the development of different antifouling strategies [7,8].

Fouling reduction has been attempted through the use of detergents and acid-base mixtures to clean the membranes, or employing techniques such as black-flushing, crossflow feed flow, ultrasound-assisted vibration, aiming to overcome this issue with the least process disturbance [9,10,11]. Recently, in-line coagulation and feed treatments were shown to be successful strategies able to minimize the effect of fouling during operation [12,13]. However, these methodologies increase the overall operational cost. Another approach to attenuate fouling concerns the control of solute-membrane interactions through the modification of physicochemical properties of the membrane surface [14,15]. In this respect, magnetic responsive membranes appear as an alternative to traditional membranes to overcome fouling [16,17,18]. Such membranes are prepared through the incorporation of magneto-responsive components, such as iron oxide-based nanoparticles (NPs), at the surface or within the membrane matrix. The presence of these magnetic-responsive components has proved to allow for a reversible adjustment of the physicochemical properties of the membranes, which translate into tunable permeate fluxes and superior antifouling characteristics. Moradian and coworkers [19] fabricated Fe_3_O_4_-incorporated polyethersulfone membranes using different coatings such as polyaniline and multiwalled carbon nanotube. The membranes were casted in the presence of 1 T magnetic field, forming a thin Fe_3_O_4_ NPs layer. The incorporation of iron oxide particles increased the overall hydrophilicity of the membranes, thereby providing excellent antifouling properties along with superior permeate fluxes under magnetic field conditions during whey protein separation.

Vankelecom and coworkers [20] developed a magnetic responsive enzyme membrane bioreactor by the dispersion of enzymatically active magnetic particles in the membrane surface. The magnetic field led to an increase in the membrane pore size, thereby decreasing the filtration resistance by 75% while preventing fouling by in situ enzymatic cleaning. Later, Giorno and coworkers [17] extended this work for the hydrolysis of pectin using a magnetic biocatalytic membrane bioreactor. This system showed the reduction in fouling represented by a decline in the membrane foulants interaction under the magnetic field. Mehrnia et al. [21] developed a magnetic membrane bioreactor comprising a polysulfone ultrafiltration membrane containing magnetic NPs with sizes ranging 60–70 nm (0.11 wt.% loading). A 40–160 mT magnetic field was applied across the membrane during operation. The magnetic-induced changes of the membrane properties caused a 68% decrease in filtration resistance, followed by a 30% increase in the permeate flux, ultimately leading to a 34% increase in chemical oxygen demand (COD) removal.

In our previous works [22,23], we developed hydrophilic nanocomposite membranes using polymeric particles with different architectures, such as spheres, worms, and vesicles, incorporating magnetic hybrid nanoparticles at the surface. These hybrid nanoparticles consisted of polymer coated iron oxide [22,23,24,25] or silver cores [26], synthesized via polymerization-induced self-assembly (PISA). The magnetic field had a positive effect, leading to a 29% increase of the hydraulic transmembrane flux ascribed to irreversible structural changes at the membrane top layer caused by the magnetic induced mobility of the NPs. In a more recent paper, we reported the fabrication of hydrophobic membranes [24] using a linear diblock copolymer made of methacrylic acid-methyl methacrylate and iron oxide NPs coated with different stabilizers (methacrylic acid and dimercaptosuccinic acid). The use of stabilizers effectively reduced the aggregation of iron oxide NPs as reported in the literature [27,28]. About 16.2% of water permeate flux increase was observed under the magnetic field.

The present work aims to complement the studies reported in the previous works by evaluating the impact of the magnetic field on protein transmission and fouling during the permeation of bovine serum albumin (BSA), which was used as the model protein. Furthermore, the effect of cyclic magnetic fields on the membrane process performance is evaluated, expecting a potential contribution of the dynamic magnetic field for the attenuation of membrane fouling. In this regard, protein permeation was conducted under different cyclic magnetic field configurations, OFF/ON and ON/OFF magnetic field cycles, in order to define the magnetic field strategy which may best enhance the effectiveness of the magnetic field.

## 2. Materials and Methods

### 2.1. Materials

Methacrylic acid (containing 250 ppm of MEHQ as inhibitor, 99%), methyl methacrylate (containing ≤ 30 ppm MEHQ as inhibitor, 99%), 4-cyano-4 (phenylcarbonothioylthio) pentanoic acid (>97%), 4,4′-azobis(4-cyanovaleric acid) (ACVA; 98%), 2-dimethylaminoethyl methacrylate (containing 700–1000 ppm of monomethyl ether hydro-quinone as inhibitor, 98%), methyl iodide, iron(III) chloride hexahydrate (97%, Reagent grade), iron(II) chloride tetrahydrate (≥99%), ammonium hydroxide (28%), and bovine serum albumin were purchased from Sigma-Aldrich (Dorset, United Kingdom) and used as received. NMR solvents, CD_3_OD, CDCl_3_, and D_2_O, were purchased from Eurisotop (Saint Aubin, France).

### 2.2. Membrane Fabrication and Characterization

The hydrophilic mixed matrix membranes were prepared following the method described by Upadhyaya et al. [23]. The nanocomposite membranes were prepared from spheres of poly (methacrylic acid)-b-(methyl methacrylate) (PMAA-b-PMMA) (PMAA_47_-b-PMMA_185_; Dispersity Index, Ð = 1.06, Number Average Molecular weight, Mn = 19.5 kg/mol) synthesized by the Reversible Addition-Fragmentation chain Transfer (RAFT) mediated polymerization induced self-assembly (PISA) technique. About 15 wt.% casting solution was prepared containing polymeric particles (spheres) interconnected with iron oxide nanoparticles coated with quaternized poly(2-dimethylamino) ethyl methacrylate. The casting solution was then spin-coated on microporous nylon supports. The water contact angle of the resulting membrane was found to be 46 ± 4°.

The hydrophobic mixed matrix membranes were prepared following the method described by Upadhyaya et al. [24]. The membranes made using a mixture of a linear diblock copolymer (poly(methacrylic acid)-*b*-(methyl methacrylate); PMAA_47_-*b*-PMMA_69_; Ð = 1.02 *M_n_* = 10.1 kg mol^−1^) and magnetic iron oxide nanoparticles. A well-defined linear diblock copolymer of poly(methacrylic acid)-*b*-poly (methyl methacrylate) was synthesized using RAFT mediated polymerization. The iron–oxide cores employed here were prepared using 2 different types of stabilizers: PMAA_47_ and meso-2,3-dimercaptosuccinic acid (DMSA). The membranes were prepared from casting solutions containing the diblock copolymer dissolved in THF and forming the PNPs by the addition of 0.35 mL of water containing dispersed iron oxide nanoparticles. Membranes were casted using the spin-coating method on microporous nylon supports. The average water contact angle of the membrane prepared with PMAA_47_-coated NPs and meso-2,3-dimercaptosuccinic acid was found to be 115 ± 3° and 118 ± 5°, respectively.

The membrane top surface and the membrane cross-section were observed by scanning electron microscopy (SEM) before and after filtration. The SEM images were obtained using a Hitachi S4800 (Hitachi, Tokyo, Japan) operating under 0.1 kV to 30 kV working voltage. To prepare the SEM samples, the membranes were frozen in liquid nitrogen for 10 min followed by sectioning. If the membrane is not frozen enough, the cross-section of the top layer will be destroyed because of the rigidity of the nylon film. High-Angle Annular Dark Field (HAADF) images were obtained with a Technai F30 (FEI) microscope, equipped with a Fischione HAADF detector at 300 keV working voltage, in Scanning Transmission Electron Microscopy (STEM) mode.

### 2.3. Membrane Filtration Studies

The filtration experiments were conducted in a homebuilt crossflow filtration cell (area of the membrane = 2.76 cm^2^) placed in between the magnets of a GMW Dipole Electromagnet (Model 3473–70, San Carlos, CA, USA) assuring uniform magnetic field across the membrane with strength up to 2.5 T. Before each permeation experiment, the membranes were permeated with demineralized water at transmembrane pressures, TMP, up to 4 bar (higher than the operation TMP) for 3 h to eliminate the effect of top layer compaction during the filtration process.

**Figure 1:**
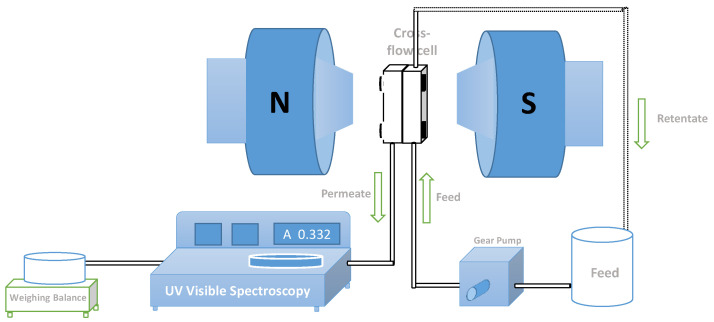
Scheme of the crossflow filtration cell between the magnetic poles with on-line monitoring of protein concentration in the permeate stream by coupling inline a UV-Visible spectrophotometer.

The performance of the membranes was evaluated by permeation of 0.5 g/L BSA solutions at pH 7.1 (feed volume of 2.35 L) at TMP of 0.5 bar and 3 bar, in the absence and presence of a magnetic field strength of 1.15 T, for 25 h. The magnetic field intensity of 1.15 T was the maximum allowed by the electromagnet considering the minimum distance between electromagnet poles required to couple the permeation cell. The permeation experiments at different TMPs were performed independently using new membranes in each case. The permeate outlet of the crossflow cell was connected to a flowthrough quartz cuvette placed in a UV-Vis spectrophotometer (Helios Alpha, ThermoUnicam, Porto, Portugal) for the on-line acquisition of permeate absorbance at a wavelength, λ, of 280 nm (Figure 1). The absorbance of permeates was then converted into BSA concentration through the Lambert-Beer equation. The permeate stream was collected in a reservoir placed on a balance connected to the SartoConnect software for data acquisition and posterior determination of the permeate fluxes. The temperature was frequently measured, and it was observed to keep a constant value of 25.3 °C throughout the whole experiment time. The permeate flux and permeability were calculated using Equations (1) and (2), where V_p_ is the permeate volume collected, t is the time, and S is the surface area.

(1) $$\mathrm{Flux}\;\left(J_{V}\right)=V_{P}/\left(t\times S\right)$$

(2) $$\mathrm{Permeability}\;\left(L_{P}\right)={\;J}_{v}/\;\Delta P\;$$

where the pressure difference ΔP is expressed by:

(3) ΔP=TMP−Δπ (bar)

where TMP is the transmembrane pressure and Δπ is the osmotic pressure difference, which was assumed to be negligible in the present studies.

Protein permeation was carried out under OFF/ON (strategy 1) and ON/OFF (strategy 2) magnetic field cycles, accomplished by sequential switches of the magnetic field between 1.15 T and 0 T. During strategy 1, the experiment was started in the absence of a magnetic field (magnetic field OFF). The magnetic field was switched ON after 6 h of operation, when a significant permeate flux decrease, due to fouling, was already observed. After this initial stage, the magnetic field was switched OFF/ON each 4 h period. The cyclic frequency was selected based on the time needed to clearly observe significant permeate flux reduction, in a way to allow for a clear perception of the magnetic field effect. In strategy 2, the permeation experiments were started in the presence of 1.15 T magnetic field, which was then switched ON/OFF each 4 h period. The protein deposited at the membrane was calculated by mass balance, taking into account the concentration of protein in permeate and retentate streams.

The apparent sieving coefficient, S_a_, was calculated based on Equation (4). S_a_ was calculated using the absorbance readings of the retentate stream every 30 min to 1 h using a UV-visible spectrophotometer (Helios Alpha, ThermoUnicam, Porto, Portugal).

(4) $$S_{a}=\frac{C_{p}}{C_{R}}$$

where C_P_ and C_R_ correspond to the concentration of BSA in permeate and retentate, respectively.

## 3. Results and Discussion

### 3.1. Effect of the Magnetic Field on the Performance of the Membranes Prepared by Supramolecular Assembly PISA and NIPS Techniques

**Figure 2:**
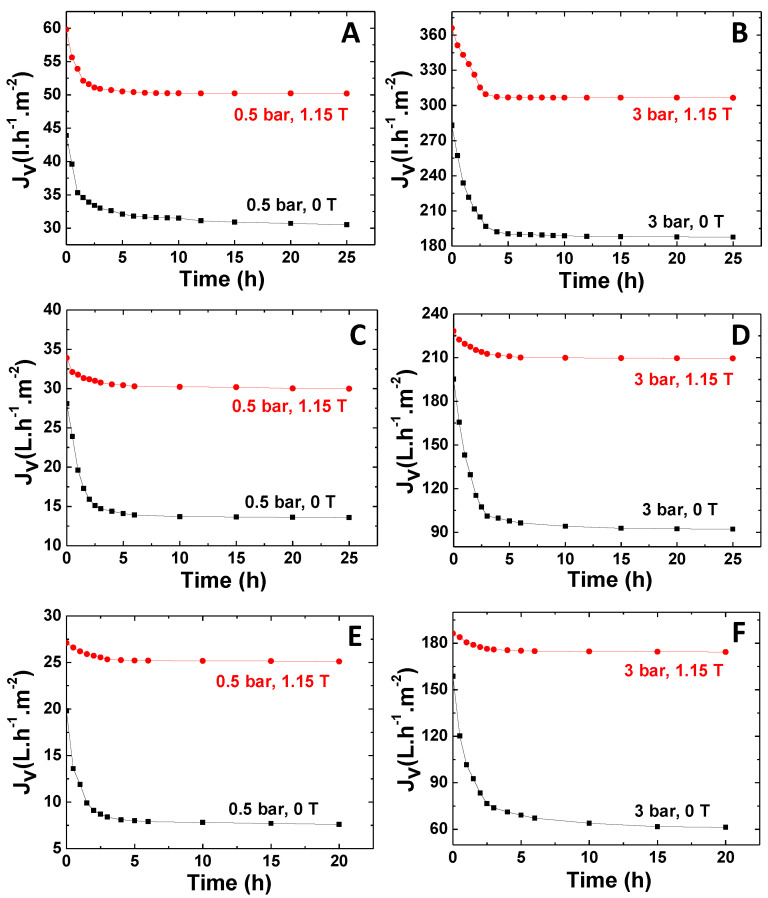
Variation of the permeate flux over time obtained during the permeation of a 0.5g/L bovine serum albumin (BSA) solution (pH 7.1) at transmembrane pressures (TMP) of 0.5 bar and 3 bar with magnetic strength of 0 T and 1.15 T, at T = 298 K, through (**A**,**B**) membranes made out of spheres prepared by the polymerization-induced self-assembly (PISA) strategy. (**C**,**D**) Membranes obtained from the nonsolvent-induced phase separation (NIPS) procedure containing PMAA_47_-coated nanoparticles (NPs), and (**E**,**F**) membranes obtained from the NIPS procedure containing DMSA-coated NPs.

The impact of the magnetic response on the permeation of a 0.5 g/L BSA solution, at transmembrane pressures (TMP) of 0.5 bar and 3 bar, through the membranes prepared using PISA-based spherical NPs (PISA membranes) and the membranes prepared by NIPS technique (NIPS membranes), is shown in Figure 2 (detailed membrane characteristics are provided in Table S1 in Supplementary Material). Here, the membranes operated at 0 T were considered as the controls, since the membranes without inorganic NPs were not stable under pressure showing intrusion into support due to lesser mechanical stability as reported in our previous work [23].

The permeate flux exhibited an identical profile for all membranes at the different TMPs tested in the absence of magnetic field (0 T), i.e., the permeate flux showed a steep decrease in the initial process stage (~2.5 h of operation), then tending to plateau after this period. The fast decline of the permeate fluxes is ascribed to the presence of concentration polarization effects and/or membrane fouling caused by protein accumulation in the membrane. A comparative analysis of the permeate flux profiles obtained for the different membranes in the absence of magnetic field showed identical declining slopes along the initial process stage (2.5 h) for processes conducted at the same TMP. However, it translated into a higher percentage decrease of the permeate flux obtained for NIPS membranes, as they exhibited lower permeate fluxes, revealing a more pronounced effect of concentration polarization and/or fouling in this case. The decrease of the permeate flux observed in the absence of magnetic field for membranes prepared by the NIPS technique was higher than 50%, whereas a permeate flux decline slightly superior to 30% was observed for membranes prepared by PISA techniques under the same magnetic field conditions.

The differences in the permeate fluxes found for PISA and NIPS membranes may be ascribed to differences in the structural and physicochemical characteristics of these membranes. The PISA membranes showed thinner top layers with organized pore structures, creating interparticle spaces in the range of the tenths of nanometers, which defined the pore size of the membrane top layer [23]. These membranes were hydrophilic, displaying water contact angles of 46 ± 4° [23]. In contrast, NIPS membranes were characterized by a hierarchical pore structure, with sizes ranging from 32 to 400 nm [24], formed during the phase separation process along with a denser cross-section.

However, NIPS membranes showed an accentuated hydrophobic degree, i.e., NIPS membranes containing DMSA-coated NPs and PMAA_47_-coated NPs depicted water contact angles of 118 ± 5° and 115 ± 3°, respectively [24], which justifies the lower permeate fluxes obtained with these membranes.

**Figure 3:**
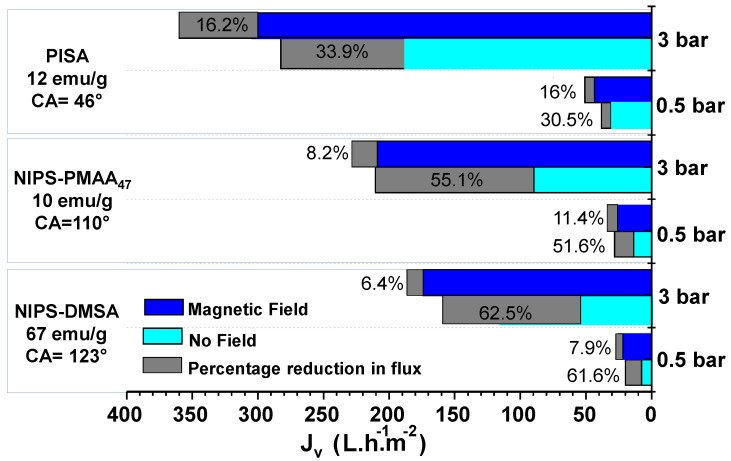
Graphical representation of initial and final permeate fluxes with or without the magnetic field for the membranes prepared by the Polymerization Induced Self-Assembly (PISA) and Non-solvent Induced Phase Separation (NIPS) techniques.

The analysis of the permeate fluxes obtained at 0 T and 1.15 T showed that the magnetic field significantly attenuates the permeate flux decline, leading to higher final permeate fluxes. The effect of the magnetic field was observed for all membranes studied, but it was more significant for the hydrophobic NIPS membranes. These differences are clearly shown in the diagram in Figure 3.

The permeation of BSA solution through PISA membranes led to a decrease in the permeate fluxes in the range of 30.5% to 33.9% at 0 T, whereas a smaller permeate flux declines of ca. 16% was observed at 1.15 T.

However, the permeation of an identical protein solution using the NIPS membranes (NIPS-PMAA_47_ and NIPS-DMSA) resulted in a decrease of the permeate fluxes, of 55.1% and 62.5%, at 0 T, but a permeate flux decay smaller than 11.5% in the presence of a 1.15 T magnetic field. In the case of NIPS membranes, it was observed that the effect of the magnetic field was more evident at higher TMPs, whereas in PISA membranes, the impact of TMP on the effect of magnetic field was irrelevant. Table 1 describes the ratio of the permeate flux decays in the absence and presence of the magnetic field, R_F_, observed at 0.5 bar and 3 bar for the different membranes studied, defined by:

(5) $$R_{F}=\frac{\Delta J_{V_{\mathrm{OFF}}}}{\Delta J_{V_{\mathrm{ON}}}}$$

where $\Delta J_{V_{\mathrm{OFF}}}$ and $\Delta J_{V_{\mathrm{ON}}}$ correspond to the variation of the permeate fluxes during the permeation process, in the absence and presence of a magnetic field, respectively.

Identical R_F_ values were obtained for processes conducted with PISA membranes at different TMPs, showing that, in this case, the magnetic field effect was independent from the TMP applied. However, this result constrasted with that observed for NIPS membranes. In this case, R_F_ clearly increased with the increase of TMP, as expressed by the increase of R_F,_ from 4.53 to 6.72 (an increase of 48.3%) and from 7.79 to 9.76 (an increase of 25.3%) when the TMP was varied from 0.5 bar to 3 bar, for permeation processes conducted with NIPS PMAA_47_ and NIPS DMSA membranes, respectively.

The ability of the magnetic field to attenuate the decrease of permeate flux during protein permeation suggests the influence of the magnetic field on the concentration polarization and/or membrane fouling phenomena.

The effect of the magnetic field on the membrane performance may be explained based on the differences in the membranes’ magnetic responsiveness, which may be associated to the distinct saturation magnetization of the membranes and by structural changes taking place at the membrane top layer due to the magnetic mobility of NPs. NIPS membranes holding DMSA-coated NPs have the highest saturation magnetization, with a value of 67 emu/g [24]. Also, NIPS membranes with DMSA-coated NPs have larger pore sizes compared to that of NIPS membranes with PMAA_47_-coated NPs (Table S1, Supplementary Material). Due to their magnetic susceptibility, the NPs move and aggregate in the presence of an external magnetic field, affecting the overall pore size. As shown in the previous work [25], the magnetic field induces the restructuration of the membrane top layer, leading to the formation of larger pores. Since the saturation magnetization of membranes with DMSA-coated NPs was higher, the movement of the NPs was expected to be higher justifying a higher destructuration of the membrane top layer, generating the formation of larger pores causing an effortless penetration of BSA molecules and entrapment into the membrane pores, resulting in a higher fouling resistance.

Although the magnetic-induced restructuration of the membrane top layer may fully explain the magnetic behavior of the permeate flux, a possible contribution of the magnetic susceptibility of BSA cannot be excluded. Permanent magnetic field has previously been reported to increase the permeate fluxes of nonmagnetic polysulphone (PS) membrane during protein permeation [29]. The increase of permeate flux in the presence of the magnetic field was attributed to lower fouling effects, evidencing, in this case, a possible role of the magnetic sensitivity of BSA on the permeation process.

However, these results seem contradictory with the effect of magnetic field on proteins’ structure and physicochemical characteristics, which was reported to favor protein intra- and intermolecular interactions. This effect is ascribed to the influence of the magnetic field on the secondary structure of proteins, with changes in the bond angles resulting from the preferential orientation of the molecular dipoles [30], which would more expectedly lead to the increase of protein interactions to the membranes than to a decrease of membrane fouling as observed.

### 3.2. Membrane Performance Under Cyclic Variation of the Magnetic Field

**Figure 4:**
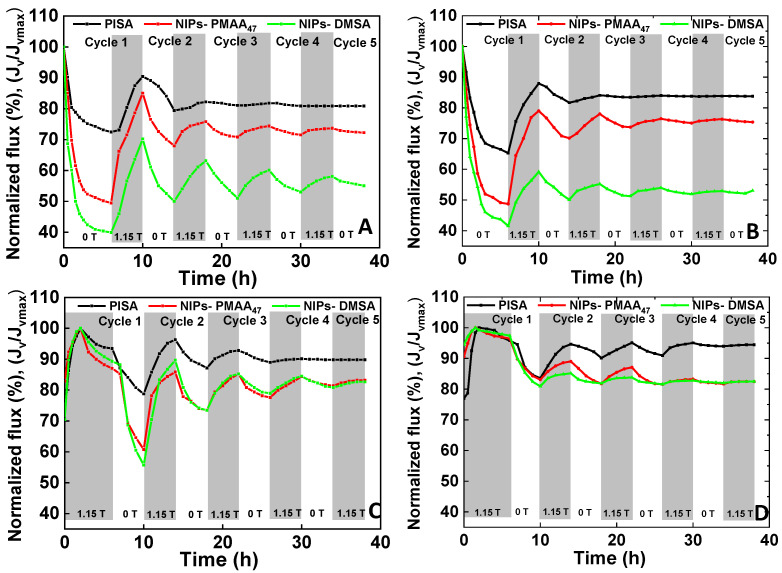
Effect of magnetic field cycles on the permeate fluxes, obtained along with the permeation of a 0.5g/L BSA solution at pH 7.1: (**A**) Strategy 1 (OFF/ON), TMP of 0.5 bar; (**B**) Strategy 1 (OFF/ON), TMP of 3 bar; (**C**) Strategy 2 (ON/OFF), TMP of 0.5 bar; and (**D**) Strategy 2 (ON/OFF), TMP of 3 bar.

To understand the reversibility of the magnetic responsiveness of the membrane performance, permeation experiments were carried out in the presence of cyclic magnetic field conditions, periodically switched between 0 T (OFF magnetic field) and 1.15 T (ON magnetic field) using two distinct operating strategies. In the first strategy (strategy 1), permeation was conducted using an OFF/ON magnetic field modulation sequence. In the first cycle, the experiment was carried out at OFF magnetic field conditions for 6 h followed by a 4 h period with the magnetic field ON. Subsequently, the magnetic field was switched every 4 h between OFF and ON conditions. In the second strategy (strategy 2), the ON/OFF magnetic field sequence was applied with the first cycle of 6 h with the magnetic field ON, followed by a 4 h stage with the magnetic field OFF. The following cycles were carried out by switching the magnetic field ON and OFF every 4 h. These experiments were carried out using a BSA solution to evaluate the influence of both strategies on the permeate flux (J) and, thus, on the membrane concentration polarization and/or the fouling effects (Figure 4).

Figure 4 shows the normalized permeate flux (J/J_max_) profiles obtained during five consecutive magnetic field OFF/ON (strategy 1) and ON/OFF (strategy 2) cycles. In strategy 1, all membranes showed a significant drop of the permeate flux along the first stage of operation at an OFF magnetic field condition, reaching values as low as 40% and 50% of the initial permeate flux, for NIPS DMSA and NIPS PMAA_47_ membranes, respectively. Since the membrane and protein magnetic responsiveness effects are excluded at 0 T, this decrease of permeate flux was exclusively attributed to the membrane fouling caused by the accumulation of BSA in the membrane. When the magnetic field was switched ON to 1.15 T, the permeate flux was partially recovered, reaching values of 85% and 70% of the initial permeate flux for NIPS PMAA_47_ and NIPS DMSA, respectively, and 90% for PISA membranes, upon 4 h with the magnetic field ON, at a TMP of 0.5 bar. The magnetic responsiveness of the permeate fluxes persisted along with successive magnetic field cycles. However, the magnetic responsiveness showed a decrease in the variation amplitude with the increase of the number of cycles. A comparative analysis of the permeate flux profiles obtained for the three membranes indicates that the magnetic responsiveness of the NIPS membranes was kept along with a higher number of cycles than PISA membranes. The magnetic responsiveness of the PISA membranes disappeared almost entirely after the second cycle, whereas the magnetic behavior of the NIPS membranes was visible through all the cycles. In line with this, the PISA membranes reached a steady permeate flux after two OFF/ON cycles.

Considering the permeate flux reduction obtained in the absence of the magnetic field (Figure 3), it is possible that the final permeate flux obtained under dynamic variation of the magnetic field intensity was higher than that observed in the absence of the magnetic field (Figure 4).

These results may be interpreted similarly to those obtained with a permanent magnetic field based on the magnetic behavior of the NPs at the membrane top layer. When the magnetic field is applied, the superparamagnetic NPs tend to align with the magnetic field and (re)organize themselves at the membrane top layer. This (re)organization allows a higher approximation of the NPs, prompting the formation of new NP nanoclusters, with the formation of unique and larger pores, which may explain the increase of the permeate fluxes observed at ON magnetic field conditions. The reorganization of the SPNPs in magnetic field has already been reported by other authors [25,31,32,33].

Consequently, the magnetostatic interactions among the magnetic moments of neighboring particles become significant, influencing the dynamic of the moments [34]. The agglomeration of the NPs in the presence of a magnetic field was observed in our previous work [25]. Once the magnetic field was switched OFF, it was more difficult for the new and more prominent NP clusters to rearrange to their previous state due to the restricted mobility of the aggregates in the membrane top layer. In contrast to the PISA membranes, the NIPS membranes showed a lower organization level. In this case, the NP clusters had higher freedom and the possibility to realign with the magnetic field once it was switched OFF, which, together with the higher saturation magnetization of these membranes, explains the maintenance of the structural reorganization and the capacity of these membranes for holding at increased number of magnetic field cycles.

In the case of strategy 2, since the experiments began with the magnetic field ON, the increase of permeate fluxes were observed since the outset, allowing membrane structural changes to occur from the initial stage of the process. However, after reaching a maximum value (after 2 h of operation), a decrease of the permeate flux was observed before switching the magnetic field off. It is unlikely that the sudden inversion of the permeate flux trend can be ascribed to membrane structural changes, but it may be ascribed to the membrane pore-clogging caused by the deposition of BSA on the membrane (intrapore and/or surface deposition), leading to membrane fouling. The behavior of the permeate flux observed in strategy 2 suggests the presence of an equilibrium between the two co-occurring events, i.e., the magnetic-induced structural changes and fouling caused by the deposition of proteins in the membrane. In the first 2 h of operation, the structural changes due to the magnetic field were the dominant effect, while after this period, it was superimposed by fouling, rendering the decrease of the permeate flux. Once the magnetic field was switched OFF (after 6 h of permeation), the permeate flux decline became more abrupt. The accentuated reduction of the permeate flux at this stage suggests the presence of a combined effect of membrane fouling and the partial structural recovery of the membrane top layer (i.e., reduction of the membrane pore sizes). The loss of the magneto-responsiveness was observed both in strategy 1 and 2 for the 3 membranes studied. However, in strategy 2, the magneto-responsiveness of the membranes was kept for a longer period, tending to a stable permeate flux value which was higher than the original permeate flux obtained of each respective membrane (see Figure 4C,D).

**Figure 5:**
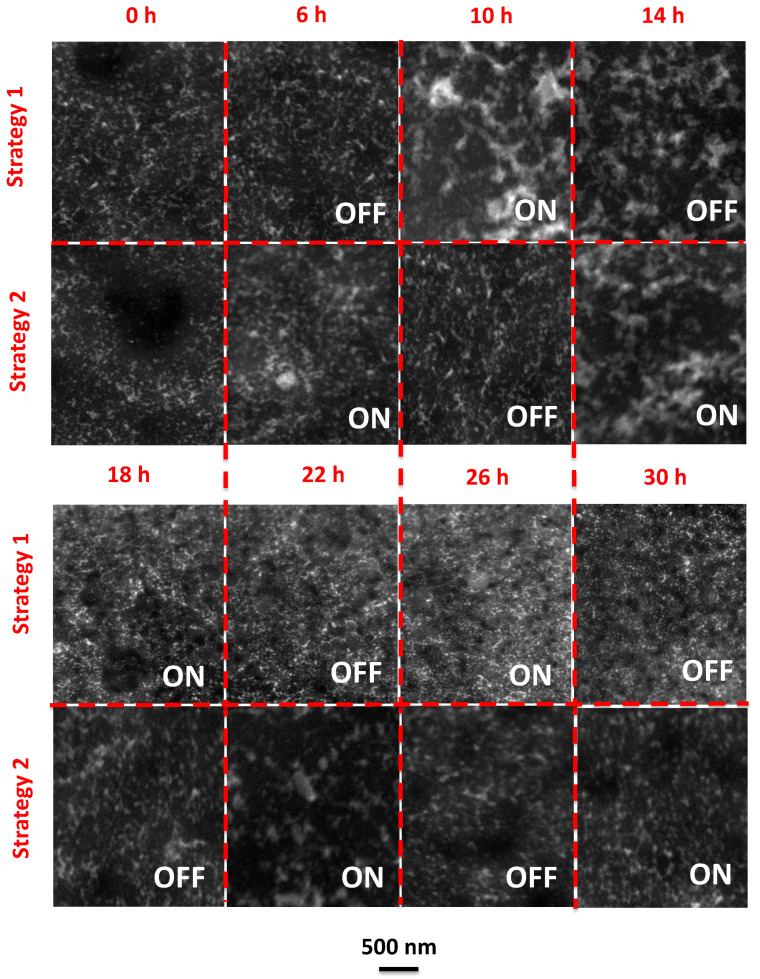
Scanning Transmission Electron Microscopy/High-Angle Annular Dark Field (STEM-HAADF) images of the sphere-like structured top layer with NPs observed for a membrane prepared by the PISA technique, when exposed to the cyclic variations of the magnetic field, according to operating strategies 1 and 2.

The reorganization and agglomeration of NPs induced by the magnetic field applied through the two different strategies were elucidated by Scanning Transmission Electron Microscopy/High-Angle Annular Dark Field (STEM-HAADF) analysis. The magnetically induced structural reorganization was simulated at a TEM grid coated with NP prepared by the PISA technique using strategy 1 and 2, respectively. In STEM mode, a very narrow electron probe is generated, followed by the sample scan, and the HAADF detector collects the electrons that were scattered at higher angles after interacting with the sample. Therefore, the elements with a higher atomic number appear with higher contrast (brighter) than lighter elements. The STEM-HAADF images obtained at different processing times are shown in Figure 5.

During strategy 1, the magnetic-induced NP agglomeration and reorganization were visible at 10 h, when the magnetic field was switched ON. The decrease in the number of NP agglomerates along the following 4 h was visible through the decrease of the amount of the higher contrast, or “white regions” in the respective STEM image. The lower amount of NP aggregates was ascribed with the partial dissociation of the NPs as a consequence of the magnetic relaxation at no field condition (14 h). No significant differences were noticed in the images acquired after 18 h of operation, confirming the inability of the membranes to change their structural properties upon the second magnetic field cycle, which is compatible with the stabilization of the membrane structure and the higher stability of the permeate fluxes attained after this operation time under strategy 1 conditions.

A different structural behavior was found for PISA membranes when exposed to strategy 2 (ON/OFF magnetic field). Analysis of the STEM-HAADF images revealed that the magnetic responsive capacity of the membranes was better preserved when they were operated using the strategy 2. Changes of the membrane structural characteristics were observed, up to at least 26 h of operation. Again, this value is in total agreement with the magnetic induced variation of the permeate flux shown in Figure 4C, which shows switches in the increasing or decreasing trend of the permeate flux up to the end of the third cycle (corresponding to 26 h of operation) at the lowest TMP (0.5 bar). These values are in agreement with the observed variation of the permeate fluxes (Figure 4), corresponding to the moments where the permeate flux stability was reached for strategy 1 and 2, respectively.

### 3.3. Impact of the Magnetic Field on Protein Permeation

**Figure 6:**
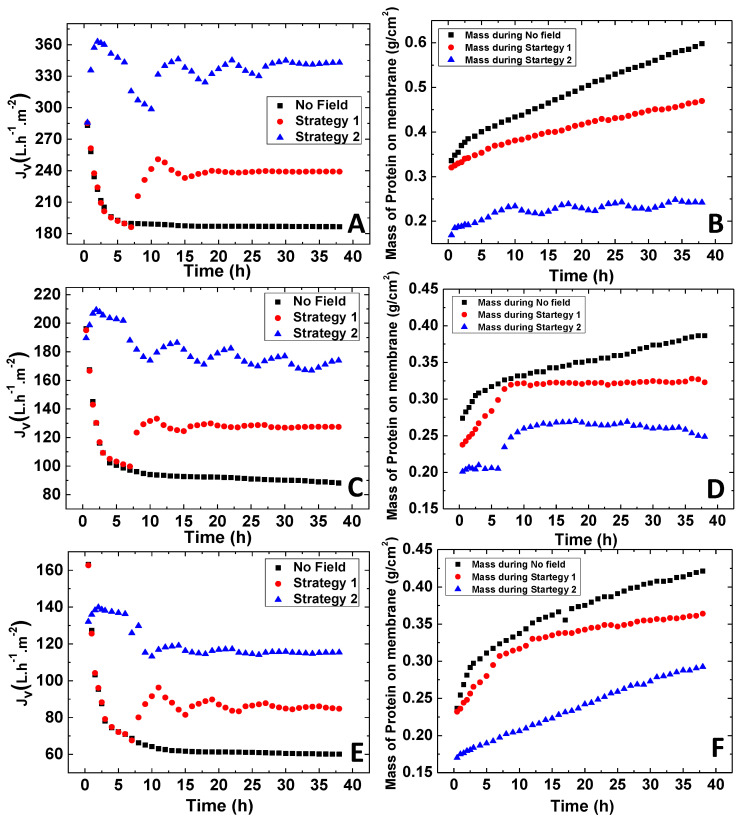
Permeate flux behavior and mass of protein retained in the membrane plotted versus time for: (**A**,**B**) Membrane made out of spheres from PISA strategy, (**C**,**D**) membrane prepared by NIPS procedure containing PMAA_47_-coated NPs, and (**E**,**F**) membrane prepared by NIPS procedure containing DMSA-coated NPs, obtained at a TMP of 3 bar.

**Figure 7:**
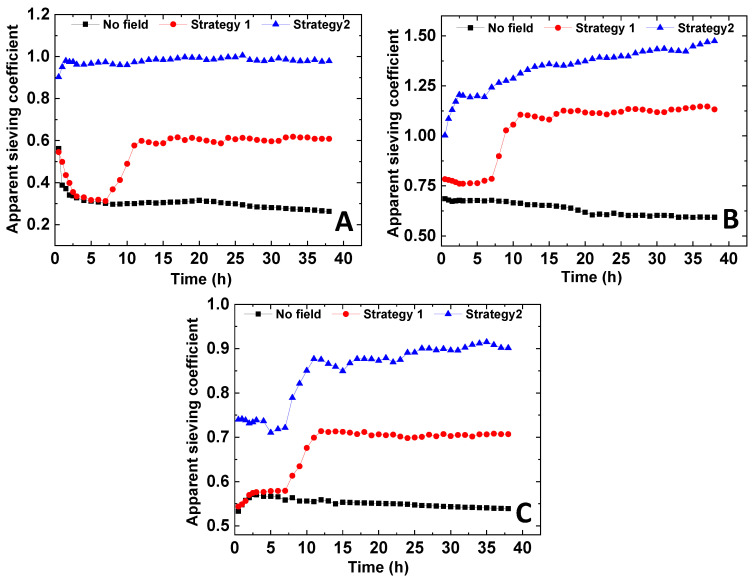
The apparent sieving coefficient versus time for (**A**) membrane holding NP spheres prepared by PISA techniques, (**B**) membranes prepared from NIPS procedure containing PMAA_47_-coated NPs, and (**C**) membranes from NIPS procedure containing DMSA-coated NPs, obtained at a TMP of 3 bar.

The behavior of the permeate flux, the mass of protein retained in the membrane during filtration, and the apparent sieving obtained for the three membranes are represented in Figure 6 and Figure 7, respectively. The results of the experiments carried out without exposure to the magnetic field and those conducted under cyclic variation of the magnetic field using strategies 1 and 2 are compared.

The correlation between the permeate flux profiles and the protein accumulation in the membranes confirmed the effect of the magnetic field for the attenuation of fouling. As expected, in all conditions, the mass of the protein in the membrane increased with time, confirming the progressive accumulation of protein in the membrane, which was more pronounced (higher membrane fouling) for the permeation experiments carried out in the absence of the magnetic field. Higher membrane fouling observed in the absence of magnetic field was followed by a continuous decrease of the apparent sieving coefficient (Figure 7), generated by a higher resistance to protein permeation.

Different apparent sieving coefficient profiles were obtained for processes operated in the presence of a magnetic field. In strategy 1, when the magnetic field was ON (after 6 h), it prompted the increase of the apparent sieving coefficient, which may be explained by the combined effect of the formation of larger membrane pores and the rise of the convective component of the permeate flux. The apparent sieving coefficients reached stability after 10 h of operation, and from there on, the sieving coefficient values remained mostly insensitive to the additional magnetic field switches.

The potential contribution of the magnetic susceptibility of BSA to the decrease of membrane fouling at magnetic field ON should be considered. However, since the protein sieving profiles were totally aligned with the magnetic behavior of the membrane structure, one may suspect that the influence of the magnetic susceptibility of BSA on the permeation process was negligible in this case.

The sieving coefficients attained for the processes conducted under strategy 2 were significantly higher than those obtained in the operations carried out at OFF/ON magnetic field cycles (strategy 1). These differences may be related to the higher magneto-responsive capacity of the membranes observed when strategy 2 was used. The presence of the magnetic field in the first process stage reduced the protein accumulation at the membrane (when compared to that occurred in strategy 1 and in the absence of magnetic field), avoiding the decrease of the apparent sieving coefficients.

Different apparent sieving profiles were also obtained with PISA and NIPS membranes when operated under strategy 2. In PISA membranes, the apparent sieving attained a nearly stable value after 2.5 h of operation (Figure 7A). In contrast, a constant increase in the apparent sieving coefficient was observed with NIPS membranes (Figure 7B,C). Again, these differences may be related to the higher magnetic responsiveness shown by the NIPS membranes (Figure 4). The NIPS membranes depicted an extended magneto-responsive capacity, meaning that the structural rearrangements were maintained for a longer period of time, prompting a higher effect in the protein transport through the membranes.

## 4. Conclusions

This work evaluated the impact of an external magnetic field on the performance of hydrophilic and hydrophobic membranes containing thin mixed matrix top layers prepared from PMAA-*b*-PMMA block copolymer with dispersed magnetic inorganic NPs during permeation of bovine serum albumin (BSA) as the model protein. The three membranes tested in this work showed magneto-responsive behavior, with an impact on the permeate fluxes and the protein sieving coefficients. The magnetic field improved the permeate fluxes by attenuation of the membrane fouling effects caused by the accumulation of protein in the membranes, and increased the apparent protein sieving coefficients, S_a_. Changes in the membrane performance were ascribed to the presence of magnetically induced structural rearrangements taking place at the membrane top layer, as reported in our previous studies [23,24,25]. Such structural rearrangements generated the increase of the pore sizes, which explained the rise of the fluid convective transport expressed by the increase of the permeate flux and the subsequent increase of the apparent protein sieving coefficient.

All membranes studied were able, to a certain extent, respond reversibly to the intermittent magnetic field, varied between 0 T and 1.15 T for both applied strategies (strategy 1: OFF/ON and strategy 2: ON/OFF sequences). The use of an ON/OFF magnetic field strategy allowed for a better preservation of the membranes’ magnetic responsiveness, possibly by avoiding membrane fouling effects during the first operation period. As confirmed by STEM-HAADF analysis, the magneto-responsiveness of these membranes was due to a magnetically induced agglomeration and disaggregation triggered by the magnetic relaxation when the magnetic field was removed. Therefore, the loss of magnetic properties of the membranes was ascribed to the progressive incapacity of NPs to disaggregate along with the cyclic variation of the magnetic field. Therefore, the use of strategies, combining magnetic field stimulation and techniques which may facilitate the NPs disaggregation, such as ultrasounds, are regarded as promising methodology to improve the magneto-responsiveness of these membranes.

Overall, magnetic mixed matrix membranes prepared by PISA and NIPS techniques showed promising results toward a better control over membrane biofouling using a non-energy-intensive magnetic field.

## Figures and Tables

**Table 1:** Ratio of permeate flux reduction, R_F_, in the absence and presence of magnetic field, obtained for membranes with different saturation magnetization, prepared by PISA and NIPS technologies.

Membrane	Saturation Magnetization (emu/g) [23,24]	The Ratio of Permeate Flux Reduction, R_F_	
		TMP = 0.5 bar	TMP = 3 bar
PISA	12	1.9	2.09
NIPS-PMAA_47_	10	4.52	6.71
NIPS-DMSA	67	7.79	9.76

TMP: Transmembrane Pressure, PISA: Polymerization Induced Self-Assembly, NIPS: Non-Solvent Induced Phase Separation, PMAA: poly (methacrylic acid), DMSA: meso-2,3-dimercaptosuccinic acid.

## Data Availability

Data available on request.

## Supplementary Materials

The following are available online at https://www.mdpi.com/2077-0375/11/2/105/s1, Table S1: Characteristics of the membranes.
