# Supplementary material for: Block Copolymer-Based Magnetic Mixed Matrix Membranes—Effect of Magnetic Field on Protein Permeation and Membrane Fouling
Source: Membranes (Basel). 2021 Feb 2;11(2):105. doi: 10.3390/membranes11020105 (PMC7912976; doi:10.3390/membranes11020105)
Supplement: Supplementary file 1 [file membranes-11-00105-s001.pdf]

membranes

MDPI

Supplementary Material: Block Copolymer Based Magnetic Mixed Matrix Membranes—Effect of Magnetic Field on Protein Permeation and Membrane Fouling

Lakshmeesha Upadhyaya, Mona Semsarilar, Damien Quemener, Rodrigo Fernández-Pacheco, Gema Martinez, Isabel M. Coelhoso, Suzana P. Nunes, João G. Crespo, Reyes Mallada and Carla A. M. Portugal

Table S1. Characteristics of the membranes.

Membrane type	Pore size (nm)	Membrane active layer Thickness (µm)	Contact angle (°)	Pure water flux[1] at 0 T ($L.h^{-1}.m^{-2}$)	Pure water flux[1] at 1.15 T ($L.h^{-1}.m^{-2}$)	Flux change (%)
**PISA membranes from spheres**	4–28	1.5	46 ± 4	375.3	485.6	29.4
**NIPS membranes containing $PMMA_{47}$-coated NPs**	60–80	5.9	115 ± 3	205.9	228.3	9.8
**NIPS membranes containing DMSA-coated NPs**	32–400	6.3	118 ± 5	155.5	186.9	16.8

[1]The permeate fluxes were obtained at a transmembrane pressure (TMP) of 3 bar. PISA: Polymerization Induced Self-Assembly; NIPS: Non-Solvent Induced Phase Separation, PMAA: poly (methacrylic acid), NP: nanoparticle; DMSA: meso-2,3-dimercaptosuccinic acid.
